# The spatial area of the normalization pool in early visual processing

**DOI:** 10.1167/jov.26.10.22

**Published:** 2026-10-22

**Authors:** Aqsa Hassan, Felix A. Wichmann, Rafal K. Mantiuk, Heiko H. Schütt

**Affiliations:** 1 Department for Behavioural and Cognitive Sciences, Université du Luxembourg, Esch-sur-Alzette, Luxembourg; 2 Neural Information Processing Group, University of Tübingen, Tübingen, Germany; 3 Department of Computer Science and Technology, Cambridge University, Cambridge, UK

**Keywords:** spatial vision, divisive normalization, contrast masking, psychophysics

## Abstract

Precisely measuring the properties of low-level visual processing is of theoretical interest as such properties often generalize to other processing in the brain and have immediate applications for image and video processing. The initial stages of human visual processing split the signal into spatial frequency and orientation-tuned channels, which interact nonlinearly, are typically modeled as divisive normalization. While we know in detail which spatial frequency and orientation channels interact, we know much less about how far the interactions reach across space. In particular, we lack psychophysical data to constrain the spatial area of the normalization pool. To provide such constraints, we set up forced choice masking experiments varying the size of the mask while we keep the spatial frequency and orientation of the mask the same as the targets. The target grating was always a small (0.5 cycles standard deviation) circular Gabor centrally superimposed over 15% and 40% contrast masks. We tested eight different mask sizes for 1, 2, and 4 cycles per degree vertical stimuli in the fovea and at 5° and 10° eccentricities. In the periphery at 5° and 10° eccentricities, contrast thresholds increase with increasing mask size and then saturate as expected, which allows us to estimate the spatial extent of the normalization pool by fitting a divisive normalization model. The fitted Gaussian normalization pool has a standard deviation of 0.65° for 1 cycle per degree gratings and scaled inversely proportional to the spatial frequency of the gratings. Neither the eccentricity nor the mask contrast influenced the size of the normalization pool. In the fovea, however, the size of the mask had no influence on thresholds, even though the mask reliably elevated thresholds. Thus, we are left with an accurate estimate of normalization pool sizes in the periphery, along with the unresolved question of why masking in the fovea shows a different behavior.

## Introduction

Understanding low-level visual processing is of both theoretical and practical interest. Low-level visual processing involves the interaction of light with the human eye, followed by the retinal encoding and the transmission of these signals through the lateral geniculate nucleus to the primary visual cortex (V1). Any stimuli that produce the same responses in the V1 after these low-level processings are perceptually indistinguishable, which is significant for image and video processing applications. In technical applications, the goal is often to reproduce stimuli that are indistinguishable from the physical world. Beyond technical applications, many of the insights we have gained about the V1 can be generalized to other areas of the brain in the higher visual pathways, making the V1 a popular testbed for theories of neural processing in general. For instance, divisive normalization was initially proposed to explain how neurons adjust their gain based on local population activity in V1, but it was later recognized as a canonical computation observed in areas as diverse as the olfactory system for odor discrimination and the parietal cortex for representing value during decision-making (Carandini & Heeger, 2012).

Neurons in area V1 and the corresponding psychophysical channels are tuned to orientation and spatial frequency, as discovered by Hubel and Wiesel (Blakemore & Campbell, 1969; Campbell & Robson, 1968; De Valois, Albrecht, & Thorell, 1982; Ferwerda, 1998; Movshon & Blakemore, 1973; Hubel & Wiesel, 1959). Groups of neurons tuned to similar frequency ranges and orientations are thought to implement the psychophysical channels. Among these channels, there are nonlinear interactions, which are the mechanism underlying center–surround suppression and contrast saturation (Xing & Heeger, 2001; Levi, Klein, & Hariharan, 2002). This mechanism is usually modeled as divisive normalization, a canonical neural computation that regulates neuronal responses by dividing the driving input of an individual neuron by the pooled activity of its neighbors (Heeger, 1992; Carandini & Heeger, 2012). Divisive normalization predicts the response of a neuron by dividing its drive by the pooled response of a set of neighboring neurons, referred to as the normalization pool. Many electrophysiological studies aimed to characterize V1 surround suppression and contextual modulation, which are assumed to be the mechanism causing the masking effects. The surrounding area that modulates a neuron’s response (the nonclassical receptive field) is estimated to have two to five times the diameter of the classical receptive field, with a mean factor of about three times and substantial variability between neurons (Cavanaugh, Bair, & Movshon, 2002a). In addition to spatial location, surround modulation depends on other properties of the surround, such as relative orientation, spatial frequency, contrast, and stimulus configuration (Series, Lorenceau, & Frégnac, 2003). It is observed that the effect of surround increases for stimuli of the same spatial frequency and orientation (Kisvárday, Toth, Rausch, & Eysel, 1997; Sengpiel, Sen, & Blakemore, 1997; Walker, Ohzawa, & Freeman, 1999). It is also found that the surrounding regions are generally asymmetric (Freeman, Ohzawa, & Walker, 2001; Walker et al., 1999). Additionally, V1 normalization can be dynamic and adaptive. Zhou, Benson, Kay, and Winawer (2019) showed that their delayed normalization model accurately accounted for various temporal phenomena, including sublinear summation, neuronal adaptation, and contrast-dependent response dynamics in human and macaque V1 neuronal responses. The normative models from natural image statistics suggest that the effective normalization pool is not necessarily a fixed, uniformly weighted region and that surround contributions may be flexibly gated according to the inferred statistical dependence between the center and the surround (Sanchez-Giraldo, Laskar, & Schwartz, 2019). Deep neural networks have also shown that responses tend to be more strongly suppressed when the surrounding features are similar to those at the center, including similarity in orientation, color, spatial frequency, and other higher-order features (Pan, DeForge, & Schwartz, 2023). Thus, there is physiological evidence that the surround of the V1 neurons behaves in more complex ways. Nonetheless, surround suppression is generally strongest for the spatial frequency and orientation that also drive the neuron most strongly, which motivates us to use a mask with the same spatial frequency and orientation as the target in our study.

Masking experiments are psychophysical studies to measure the properties of the normalization pool in human vision (Legge & Foley, 1980; Ross & Speed, 1991; Watson & Solomon, 1997; Wichmann, 1999; Bird, Henning, & Wichmann, 2002; Itti, Koch, & Braun, 2000; Levi et al., 2002). In such experiments, the observer’s task is to report which stimuli contained a target superimposed on the mask grating. The behavioral data collected through these studies allow us to constrain the nonlinear interactions between spatial frequency and orientation-tuned channels by quantifying how the contrast thresholds are modulated as a function of mask properties like mask contrast, spatial frequency, or orientation. Past studies have conducted extensive masking experiments to study the effect of many other masking stimulus parameters, and a few previous studies have changed the spatial arrangement of the target and mask, but to our knowledge none have directly measured the spatial extent of the normalization pool.

Only a few physiological studies have investigated the spatial extent of the normalization pool: Aqil, Knapen, and Dumoulin (2021) measured functional magnetic resonance imaging data and proposed a population receptive field model based on divisive normalization to capture a variety of nonlinear phenomena such as suppression, compression, and over-saturation. They modeled the activation and normalization population receptive fields as two-dimensional isotropic Gaussians that were centered on the same point in space. They found that the normalization pool was always larger or at least the same size as the activation pool. However, their size estimates for voxels in functional magnetic resonance imaging studies cannot be translated into pool sizes for individual neurons, as each voxel contains neurons tuned to different locations and spatial frequencies. Additionally, Cavanaugh et al., (2002a) and Cavanaugh, Bair, and Movshon (2002b) recorded electrophysiologically from the macaque V1 and established that the extent of the modulatory surround mechanism is approximately three times the diameter of the center mechanism, although surrounds were measured at one to five times the central receptive field size. However, neither of these studies are based on psychophysics and hence we still lack behavioral data to characterize the spatial extent of the normalization pool.

Current models would benefit from a good estimate of the normalization pool size, because they typically use pixel-wise normalization or an equal weight across all responses over the whole image (Schütt & Wichmann, 2017), although it is known from physiology that the true normalization pool is neither perfectly local nor spans the entire space. Instead, it appears to be a middle ground consisting of local groups of neurons. Additionally, it is still unknown whether the kernel that describes the interaction in divisive normalization is fixed or adaptive, that is, dependent on the stimulus (Louie, LoFaro, Webb, & Glimcher, 2014; Malo, Esteve-Taboada, & Bertalmío, 2024). The low-level vision models proposed in past research consider the size of the normalization pool a free parameter, which is fitted to specific behavioral data (Watson & Solomon, 1997; Itti et al., 2000; Mantiuk, Hanji, Ashraf, Asano, & Chapiro, 2024).

The spatial frequency and the eccentric distance from the fovea appeared to be the most plausible factors influencing the size of the normalization pool. The receptive fields of neurons tuned to higher spatial frequencies are substantially smaller (De Valois et al., 1982), and the scale of statistical structures varies with spatial frequency. Both of these factors make it plausible that the normalization pool could be different for different spatial frequencies. For eccentricity, we could expect effects due to cortical magnification, according to which a fixed distance in the cortex corresponds with a greater distance in the outside world in the periphery than in the fovea (Rovamo, Virsu, & Näsänen, 1978). Thus, we decided to test the influence of these two variables here.

This article aimed to directly investigate the spatial extent of the normalization pool by a combination of psychophysical experiments and modeling of the resulting behavioral data. We conducted a contrast masking experiment to measure how different mask sizes affect contrast thresholds. Quantifying how strongly different sizes of masks affect contrast thresholds should constrain the spatial extent of the normalization pool in divisive normalization.

## Methods

### Hardware setup

We used a BenQ X3100i DLP projector setup with a maximum luminance of 126.9 cd/m^2^ with a resolution of 128 pix/deg (3,840 × 2,160) and a refresh rate of 60 Hz. Observers were positioned at a distance of 298 cm from the 90-cm × 160-cm display screen in a darkened room with their heads positioned on a chin rest to discourage head movements. The experiment code used Psychtoolbox-3 (Kleiner, Brainard, & Pelli, 2007) and MATLAB version R2024b (MathWorks, Natick, MA). Additionally, we enabled HDR10 mode in Psychtoolbox-3 so that the display was driven by a 10-bit signal.

Nonlinearity correction was performed by measuring 17 grayscale patches sequentially displayed at the center of the screen using a Konica Minolta LS-150 luminance meter (Konica Minolta, Tokyo, Japan). The tested pixel levels were linearly spaced from 0 to 1,023 (the 10-bit maximum). To interpolate the mapping from desired luminance to RGB values, we fitted a third-degree polynomial function. We applied the color correction through the PsychColorCorrection ‘LookupTable’ in Psychtoolbox-3. Linearity correction and maximal luminance were verified by measuring the same 17 grayscale patches again after applying the ‘LookupTable’.

For spatial nonuniformity correction, we captured ‘.raw’ camera images of a uniform field displayed on the projector screen using a Nikon D810 camera (Nikon, Melville, NY). These captures were processed as 16-bit linear grayscale images and were divided by an average of three baseline camera calibration images taken of a known uniformity corrected Apple Studio display for flat-field correction. Because the camera also introduced geometric and perspective distortions, we further used OpenCV to undistort these images and applied a perspective transform to warp the screen borders onto a flat 1,920×1,080 pixel grid. To compensate for high frequency noise, a Gaussian filter (51×51 pixel kernel) was applied. The 3 captures taken at each of the equally stepped 11 luminance levels covering the 10-bit luminance range were then normalized and averaged to generate the final estimate of the two dimensional (2D) gain map. The inverse of this 2D map was applied via the PsychColorCorrection ‘GainMatrix’ functionality in Psychtoolbox-3, where the gain matrix was calculated by dividing 0.8 by the 2D gain map. The correction factors ranged from a minimum of approximately 0.78 near the center to a maximum of 1.22 in the corners. Substantial adjustments were primarily applied in the peripheral regions of the screen, particularly at the corners, where higher gain factors were used to counteract luminance fall-off and maintain a uniform luminance across the full display area. After correction, luminance was constant over the entire screen area as informally checked with the luminance meter.

### Participants

Ten naïve observers were initially recruited, of whom three participants were excluded. These three participants did not meet the selection criteria for no visual impairment and normal or corrected-to-normal visual acuity (at least 6/6), tested using the Snellen eye chart. The remaining seven observers participated in the full experiment. This study was conducted in accordance with the declaration of Helsinki and the guidelines of the University of Luxembourg, Luxembourg, and the European Union as reviewed and approved by the Ethics Review Panel (ERP) of the University of Luxembourg (Approval No. ERP 24-090 VisDisc). Participants gave informed consent and were compensated with vouchers at a rate of €20 per hour.

### Stimuli

All stimuli were vertical sinusoidal gratings with the same spatial phase such that targets and masks differed only in their spatial envelope and contrast levels. Example stimuli used in our experiment are given in Figure 1.

**Figure 1.**
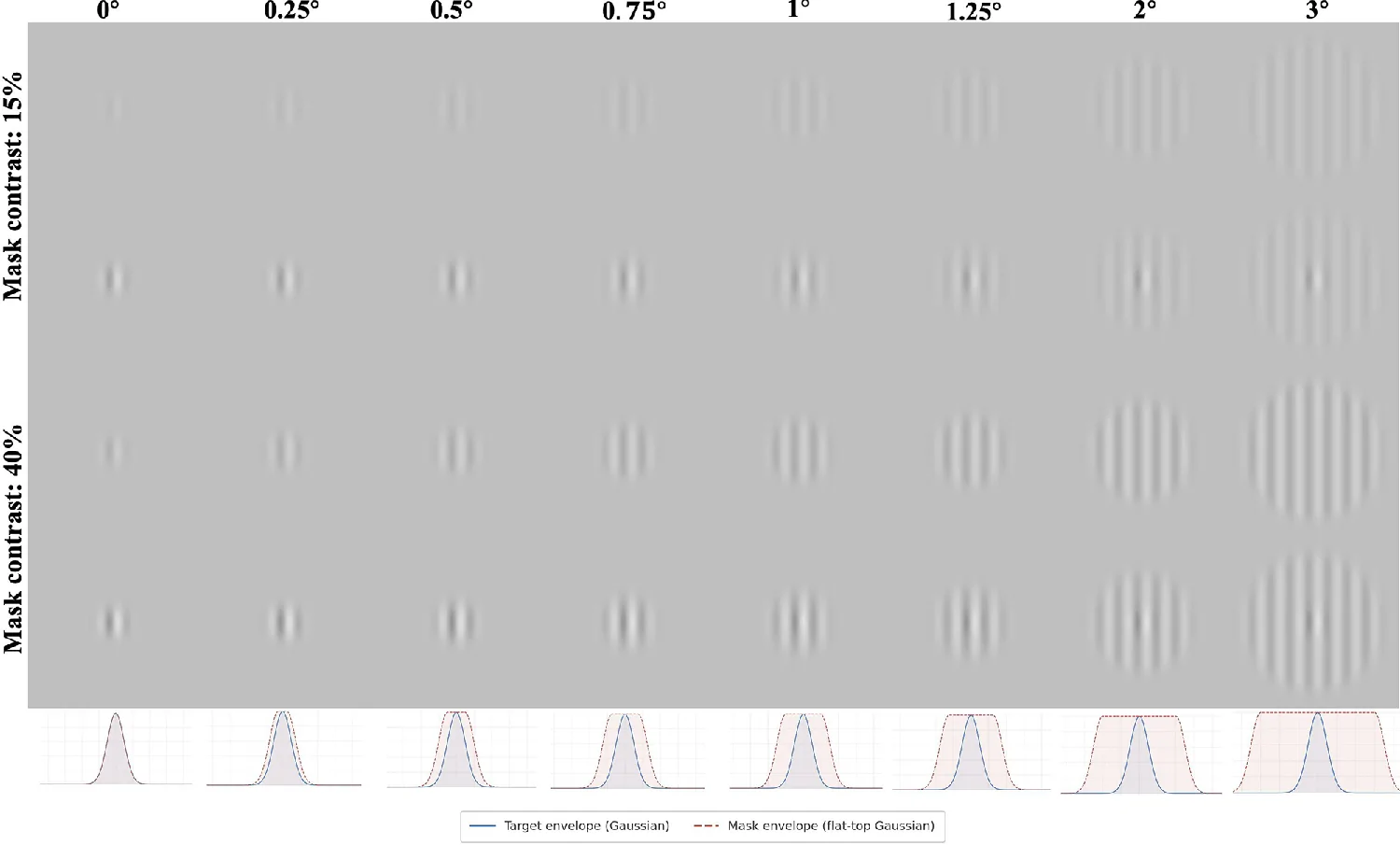
Example stimuli for a spatial frequency of 1 cpd. For demonstration purposes, the target contrast is 55%. Columns correspond with the mask sizes. The first row presents only the mask grating at 15% contrast, and the second row shows the target grating superimposed on this mask. Similarly, the third row shows the mask grating alone at 40% contrast, and the fourth row presents the target grating superimposed on this 40% contrast mask grating. The last row demonstrates the target and mask envelopes for each mask size.

The targets were sine phase Gabor patches generated by multiplying a sinusoidal grating with a Gaussian envelope. The targets had a standard deviation of 0.5 cycles and were centrally superimposed over the 15% and 40% contrast masks. The masks were produced by multiplying a sinusoidal grating with a “flat-top” envelope *E*_mask_(*r*) as given in Equation 1:

(1) $$\begin{array}{c} \;E_{\text{mask}}\left(r\right)=\left\{\begin{array}{cc} \;1 & ;0\leq r<\text{mask}\;\text{size}\\ \exp\;\left[\;-\frac{{\left[r-\text{mask}\;\text{size}\right]}^{2}}{2\sigma_{\text{target}}^{2}}\;\right] & ;\text{mask}\;\text{size}\leq r\leq \text{mask}\;\text{size}+3\sigma_{\text{target}},\\ \;0 & ;r>\text{mask}\;\text{size}+3\sigma_{\text{target}} \end{array}\right.\; \end{array}$$

where σ_*target*_ is the standard deviation of the target, which is equal to 0.5/*f*, and *r* is the Euclidean distance from the center of the stimulus.

This circular symmetric envelope of the mask was composed of a circle of constant maximal contrast whose radius is the nominal mask size, surrounded by a gradual border with the same fall-off as the Gabor target, as demonstrated in Figure 1. At a nominal mask size of 0, the envelope becomes the target’s envelope, such that the task effectively becomes the standard contrast discrimination with the signal and the mask perfectly coincidental in space. We used eight different mask sizes to study the spatial extent of the normalization pool: 0°, 0.25°, 0.50°, 0.75°, 1.00°, 1.25°, 2.00°, and 3.00°. The stimulus centers were displayed at 0°, 5°, or 10° eccentricity along the vertical center line of the projector screen. We tested 1, 2, and 4 cycles per degree (cpd) stimuli.

### Procedure

The experimental design used two forced choice paradigms: the two alternative forced choice (2AFC) paradigm for stimuli displayed at the parafovea and the two interval forced choice (2IFC) paraigm for stimuli displayed at the fovea. Stimuli were presented for 200 ms, that is, 12 frames at a 60-Hz frame rate. For the 2AFC task at eccentricities of 5° and 10°, the mask grating was displayed on both sides of a central fixation dot, while the target grating was superimposed on the mask grating only on one randomly chosen side. In the 2IFC foveal task, the mask grating was presented at the center of the screen in both intervals, while the target was superimposed on only one of them. These two stimulus intervals were separated by a 200-ms intermediate blank interval. In both tasks, the response interval was indicated by a green fixation dot shown at the center of the screen, and observers were given 1,000 ms to respond using the keyboard. The observers’ task was to discriminate on which side or interval the target appeared.

The contrast of the target patch was adaptively chosen from a set of 100 contrast levels between 0% and 55% using a one-step-look-ahead procedure to minimize the expected entropy of the posterior over threshold and width of the psychometric function (Kontsevich and Tyler, 1999; Prins, 2013). The posterior was sampled using a grid of 40 values for the threshold, 20 for the width, and 10 for the lapse rate, which was adapted whenever a boundary point reached a posterior probability of less than 1/10,000.

Each block consisted of 100 trials. Each observer performed a total of 144 blocks divided into 10 one-hour sessions with 12 to 16 blocks per session. The observer performed 96 blocks of the parafoveal 2AFC task consisting of combination of 8 mask sizes, 2 eccentricities, 2 mask contrasts, and 3 spatial frequencies. The remaining 48 blocks of the 2IFC task with combination of 8 mask sizes, 2 mask contrasts, and, 3 spatial frequencies displayed in the fovea. Observers were allowed to take short breaks between blocks. They also performed a short training before each session with two blocks of 100 trials each in which they discriminated the luminance of blobs to familiarize themselves with the task and keyboard controls.

Data from each individual block were fit with a Weibull function using psignifit 4 (Schütt, Harmeling, Macke, & Wichmann, 2015) defining the middle point between the guess rate and the upper asymptote as the threshold. The estimated psychometric widths on a logarithmic scale remained rather uncertain and did not show any clear pattern across conditions. They remained constant at about 1.3 logarithmic contrast.

## Results: Psychophysics

Figure 2 summarizes the averaged contrast thresholds for all observers. In this plot, the error bars are estimated as 95% confidence intervals based on a t-distribution with the standard error of the mean (SEM) between observers. The logarithmic contrast thresholds are given on the *y*-axis, which are taken to be the 75% correct performance level of the Weibull cumulative distribution. It can be observed in these plots that the 40% mask contrast yielded higher thresholds than the 15% mask contrast, consistent with the vast literature on contrast masking (Legge & Foley, 1980; Itti et al., 2000). Furthermore, higher spatial frequencies also resulted in increased contrast thresholds, a result that again agrees with the existing research. For instance, it is found that reducing the size of the target stimulus with increasing spatial frequency generally yields low-pass shapes for the contrast sensitivity function (Rovamo, Luntinen, & Näsänen, 1993). At equal size, one would expect a peak at some eccentricity dependent spatial frequency (Campbell & Robson, 1968; Kelly, 1979; Mantiuk, Ashraf, & Chapiro, 2022).

**Figure 2.**
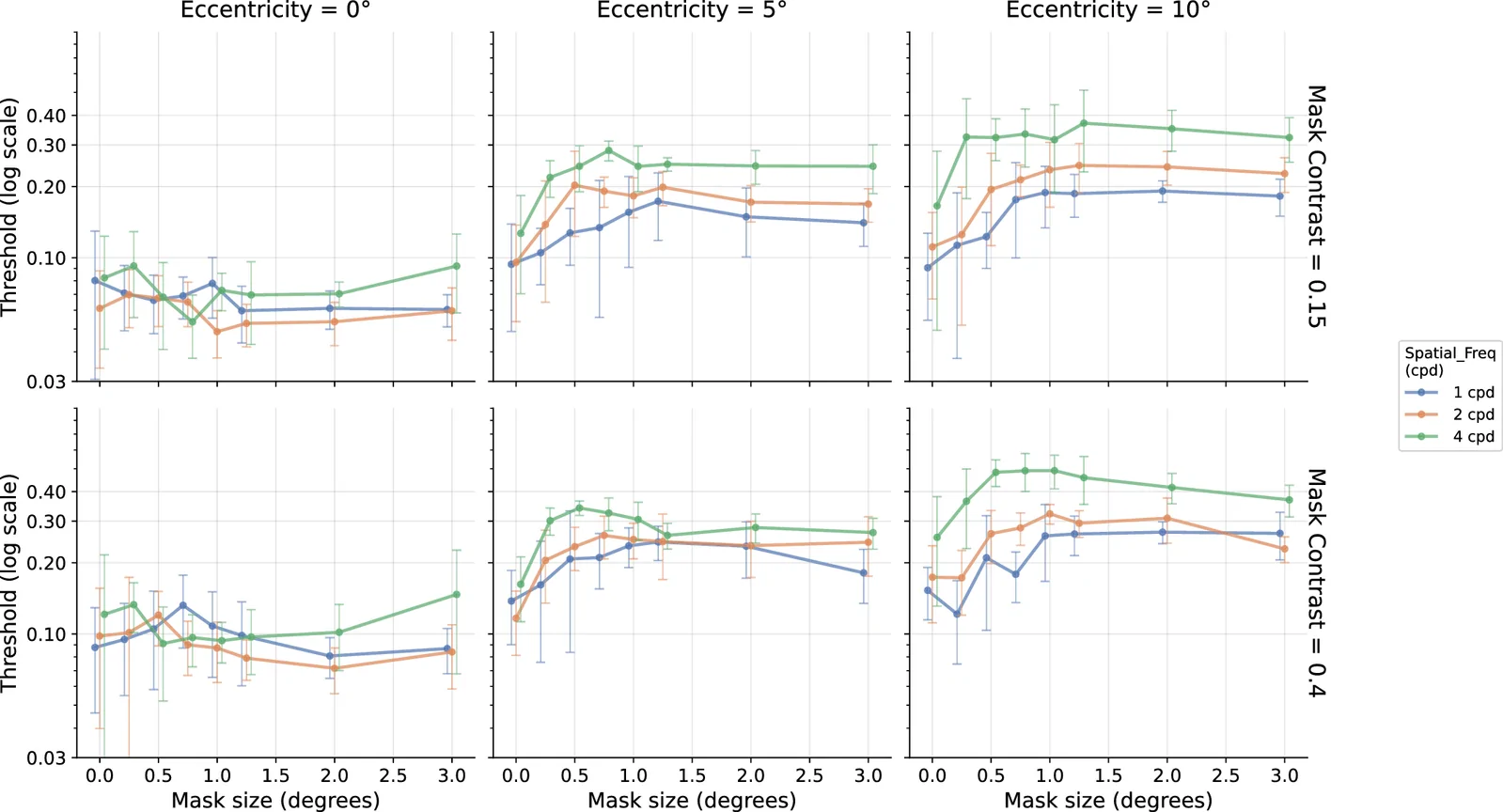
Average thresholds across observers. Error bars represent 95% confidence intervals based on a *t*-distribution with the SEM. The logarithmic contrast thresholds on the *y*-axis are taken to be the 75% correct performance level of the Weibull cumulative distribution. The *x*-axis consists of the mask sizes in visual degrees.

At least for the 5° and 10° eccentricities, contrast thresholds increase with increasing mask size and then saturate, which can be useful in determining the spatial extent of the normalization. It should be noted that the curves at higher spatial frequencies (i.e., 4 cpd) are steeper than those at lower spatial frequencies (i.e., 1 and 2 cpd). This finding suggests that channels for higher spatial frequencies have normalization pools of a smaller spatial area than those tuned to lower spatial frequencies. In addition, the shape of the masking curve appears to be quite similar for the different spatial frequencies shown in Figure 3. These curves are normalized by subtracting their mean thresholds in log scale, and the mask sizes are expressed as the number of cycles. It can be observed in these normalized curves that the shape of the contrast-masking curve is independent of the spatial frequencies.

**Figure 3.**
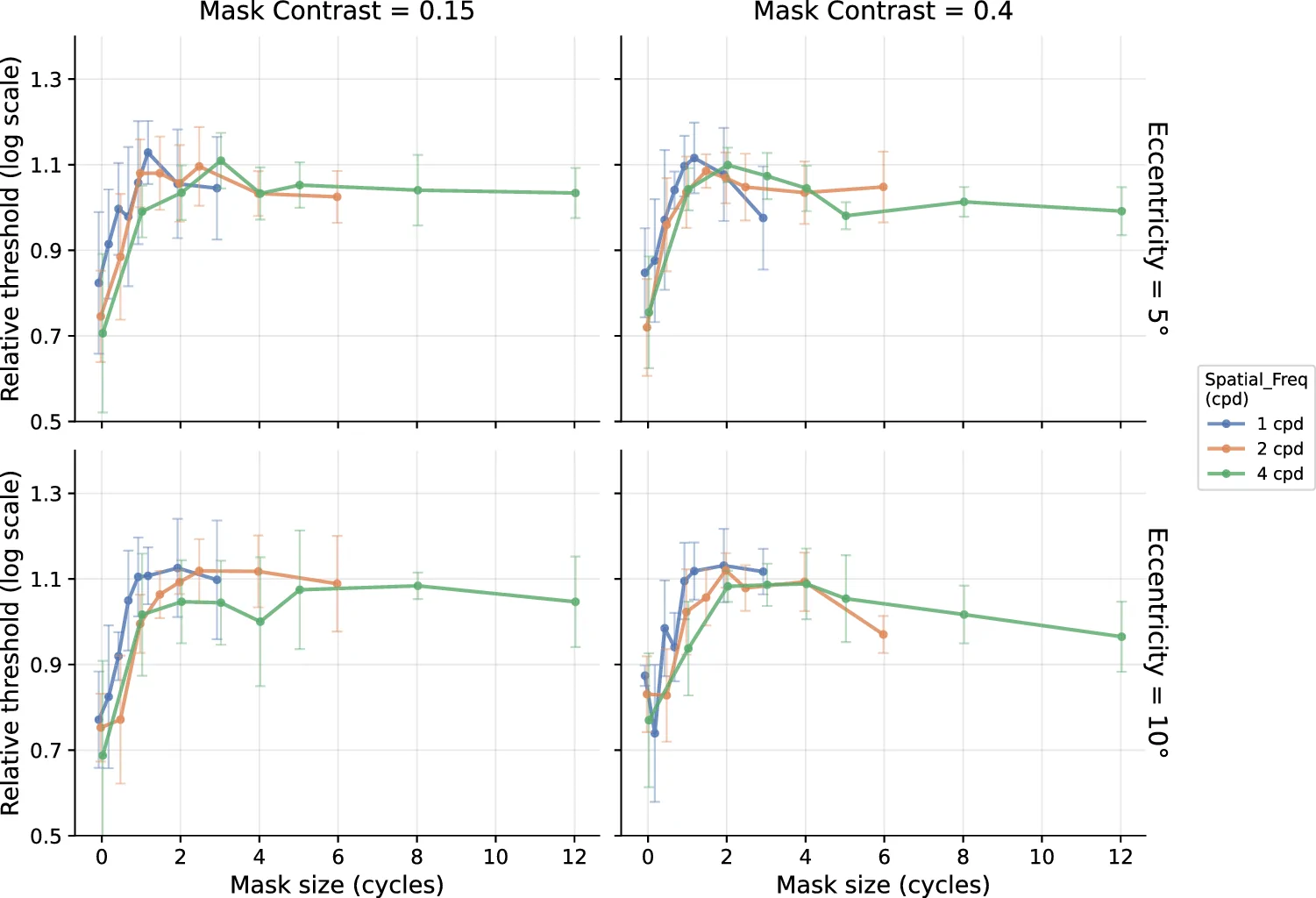
In these plots, the contrast thresholds are normalized by subtracting their mean thresholds in log scale and the mask sizes are expressed as the number of cycles. It can be observed that the shape of the masking curve is independent of the spatial frequency.

In the fovea at 0° eccentricity, the mask size has a minimal effect on the contrast thresholds. Although detection thresholds were not measured for our observers in this study, these contrast-masking thresholds are substantially higher than the typical contrast detection thresholds previously reported in the literature at comparable spatial frequencies of 1, 2, and 4 cpd (Campbell & Robson, 1968; Owsley, Sekuler, & Siemsen, 1983; Ashraf, Wuerger, Kim, Martinovic, & Mantiuk, 2020). Moreover, increasing the contrast of the masking gratings from 15% to 40% increased the mean thresholds by a factor of 1.4, which itself is evidence that masking is present in the fovea. In the Discussion, we discuss some potential explanations for this unexpected result. In either case, these results do not allow us to estimate a normalization pool size for the foveal condition.

## Modeling

We fit a divisive normalization model (Heeger, 1992; Carandini & Heeger, 2012) to our data to estimate the size of the normalization pool. To predict which stimuli were distinguishable to our observers, we modeled the response of a single channel tuned to the eccentricity, spatial frequency, and orientation of the target. According to the divisive normalization model, this response is proportional to the ratio of the input drive *D*_0_ raised to a power *p* + *q*, divided by a weighted sum of these drives over a pool of similarly tuned neurons $\sum_{k}w_{k}D_{k}^{p}$ (the normalization pool) as given in Equation 2:

(2) $$\begin{array}{c} R=\gamma \frac{D_{0}^{p+q}}{\beta_{s}^{p}+\sum_{k}w_{k}D_{k}^{p}}\;,\; \end{array}$$

where the exponent in the numerator is larger by the parameter *q*, which is a common implementation in psychophysical models to capture that the channel contains neurons with different half-saturation constants (Watson & Solomon, 1997). In this equation, γ determines the overall scale of the response, β_*s*_ is a scaling parameter that determines how responses saturate with increasing driving input, and *p* and *q* are exponents that modify the shape of the nonlinearity.

To derive the drive of the channels from the contrast and size of the target and the mask, recall that the target grating was superimposed on the mask grating as shown in Figure 1. The mask grating always matched or exceeded the spatial extent of the target grating in our experiment. This differs from other stimuli commonly used to investigate center-surround interactions, which use a grating in the center and an annular grating as the surround, thereby defining a clear boundary between the center and the surround (Xing & Heeger, 2001). Thus, in our case, where the target grating is superimposed on the mask grating, the drive to the target-tuned channel is proportional to the sum of the mask contrast *c*_*m*_ and the target contrast *c*_*t*_ multiplied by the sensitivity *s* to the target spatial frequency *f* and eccentricity *e* as given in Equation 3.

(3) $$\begin{array}{c} D\propto \left(c_{m}+c_{t}\right)s\left(f,e\right)\; \end{array}$$

To compute the drive to the normalization pool in the denominator $\sum_{k}w_{k}D_{k}^{p}$, we modeled the weights of the normalization pool *g*_*norm*_(**x**, σ_*n*_) as a symmetric circular Gaussian kernel with a standard deviation σ_*n*_ and computed the drive to this as the integral over these weights with the sum of the target envelope *t* and the mask envelope *m* as shown in Equation 4.

(4) $$\begin{array}{ccc} \; & & \sum_{k}w_{k}D_{k}^{p}\propto s\left(f,e\right)\int g_{norm}\left(x,\sigma_{n}\right){\left[c_{m}m\left(x\right)+c_{t}t\left(x\right)\right]}^{p}dx.\; \end{array}$$

We can then combine these drives according to the divisive normalization formula as defined in Equation 2, which yields the mean response to a target contrast of *c*_*t*_ and a mask contrast *c*_*m*_ as given in Equation 5:

(5) $$\begin{array}{c} \;\mu_{t}=\frac{a{\left[\left(c_{m}+c_{t}\right)\;s\left(f,e\right)\right]}^{p+q}}{\beta_{s}^{p}+b{\left[s\left(f,e\right)\int g_{\text{norm}}\left(x,\sigma_{n}\right)\;\left[c_{m}m\left(x\right)+c_{t}t\left(x\right)\right]\;dx\right]}^{p}}.\; \end{array}$$

Additionally, we include the relative scaling parameters *a*, *b* here to capture the unknown proportionality constants for our two drives. As multiplying *a*, *b*, and β_*s*_ in this formula with the same factor would yield identical responses and predictions, we arbitrarily set the scale by fixing *a* = 1.

The channel response to the mask alone is estimated simply by setting *c*_*t*_ equal to zero as given in Equation 6:

(6) $$\begin{array}{c} \mu_{m}=\frac{a{\left[\left(c_{m}\right)s\left(f,e\right)\right]}^{p+q}}{\beta_{s}^{p}+b{\left[s\left(f,e\right)\int g_{\text{norm}}\left(x,\sigma_{n}\right)\left[c_{m}m\left(x\right)\right]\;dx\right]}^{p}}.\; \end{array}$$

The internal channel responses are modeled as Gaussian random variables centered on the means μ_*m*_ and μ_*t*_. Assuming a constant noise standard deviation σ_*noise*_ across all conditions, the probability that an optimal observer correctly identifies the target in a 2AFC task is given by the cumulative normal distribution as given in Equation 7 (Green, Swets et al., 1966):

(7) $$\begin{array}{c} P_{\text{opt}}\left(\text{hit}\right)=\Phi \left(\frac{\mu_{t}-\mu_{m}}{\sqrt{2}\;\sigma_{\text{noise}}}\right).\; \end{array}$$

To include the possibility that observers make errors for stimuli that they can easily discriminate, we include a lapse rate λ to compute the final probability that an observer makes the right decision as given in Equation 8:

(8) $$\begin{array}{c} P\left(\text{hit}\right)=\lambda +\left(1-2\lambda \right)\;\Phi \left(\frac{\mu_{t}-\mu_{m}}{\sqrt{2}\;\sigma_{\text{noise}}}\right).\; \end{array}$$

Table 1 summarizes the variables used in our model, along with their initialization and bounds.

**Table 1.** The description of model parameters, their initialization, and bounds are given in this table.

Symbol	Description	Initialization	Bounds
Model parameters			
λ	Lapse rate	0.02	[0, 0.1]
*p*	Excitatory exponent	2.0	[2, 5]
*q*	Inhibitory exponent	0.5	[0.1, 1.0]
β_*s*_	Semisaturation constant	0.5	[0.01, 1.50]
σ_*noise*_	Internal noise standard deviation	0.036	[10^−4^, ∞]
*a*, *b*	Relative scaling parameters	1	[10^−4^, ∞]
σ_*n*_	Standard deviation (size) of normalization pool	0.5	[0.01^†^, ∞]
*s*(*f*, *e*)	Sensitivity at freq. (*f*) and ecc. (*e*)	0.40, 0.45, 0.25, 0.35, 0.15	[0.1, 1.0]
Model variables			
*c*_*m*_	Mask contrast	—	—
*c*_*t*_	Target contrast	—	—
μ_*t*_	Mean response to target and mask	—	—
μ_*m*_	Mean response to mask alone	—	—
*P*(*hit*)	Probability of correct decision (2AFC)	—	—

† Where *s*(1, 5) and *a* were fixed to 1 during parameter estimation.

### Including a contrast sensitivity function

To capture observers’ sensitivity to the spatial frequencies at the different eccentricities, we first fit a separate parameter for each combination. Later, we incorporated stelaCSF (Mantiuk et al., 2022) into our model, which reduced the number of estimated parameters as defined in Equation 9. The stelaCSF model provides a prediction of the sensitivity to all spatial frequencies and eccentricities that we used.

(9) $$\begin{array}{ccc} & & \;\mu_{t}=\frac{a{\left[\left(c_{m}+c_{t}\right)s_{\text{stelaCSF}}\left(f,e,A\right)\right]}^{p+q}}{\beta_{s}^{p}+b{\left[s_{\text{stelaCSF}}\left(f,e,A\right)\int g_{\text{norm}}\left(x,\sigma_{n}\right)\left[c_{m}m\left(x\right)+c_{t}t\left(x\right)\right]\;dx\right]}^{p}},\;\; \end{array}$$

where *A* = π(0.5/*f*)^2^ is the area of our target grating where the radius is considered to be one standard deviation of the stimulus as defined in stelaCSF.

### Model parameter estimation

We fit our models only to the eccentric data because no substantial effect of mask size was observed in our foveal data. To estimate the parameters, we defined the probability of a hit as defined in Equation 8 and performed the maximum likelihood estimation using the Broyden–Fletcher–Goldfarb–Shanno optimizer as implemented in SciPy (Virtanen et al., 2020). The estimated parameters are as follows:

• Estimating sensitivities at measured spatial frequencies and eccentricities: *s*(1, 5), *s*(1, 10), *s*(2, 5), *s*(2, 10), *s*(4, 5), *s*(4, 10), where we arbitrarily fixed *s*(1, 5) = 1 to remove ambiguity about the scale of the internal axis. Later, rather than estimating these sensitivities, we integrated stelaCSF.
• Standard deviation of the normalization pool σ_*n*_ for each condition, that is, $\sigma_{g_{norm}}\left(1,5\right)$, $\sigma_{g_{norm}}\left(1,10\right)$, $\sigma_{g_{norm}}\left(2,5\right)$, $\sigma_{g_{norm}}\left(2,10\right)$, $\sigma_{g_{norm}}\left(4,5\right)$, $\sigma_{g_{norm}}\left(4,10\right)$, for which we set dependencies in later models.
• Exponents *p* and *q*, the half-saturation constant β_*s*_, and the relative scaling parameter *b*.
• Standard deviation of the noise in the channel σ_*noise*._
• Lapse rate λ.

### Modeling results

We compared six different variants of our proposed model in total across all conditions (see the Appendix). However, in this section, we have discussed only the two most relevant models. It includes our *preferred* model, which inversely scales the size of the normalization pool with spatial frequency while keeping it identical across the two eccentricities, that is, 5° and 10°. The other is the *separatePool* model, in which we independently estimated the normalization pool size for each combination of location and spatial frequency. The conditions used in modeling consisted of the six combinations derived from three spatial frequencies (1, 2, and 4 cpd) and two eccentricities (5° and 10°). Varying the mask contrast (15% vs. 40%) did not have a significant effect on the normalization pool. Note that the foveal measurements were intentionally excluded from the modeling analysis because the mask size has a minimal effect on these thresholds measured in fovea.

For model comparison, we performed leave-one-subject-out cross-validation and used the model with a single fixed normalization pool size as the baseline, that is, where the size of the normalization pool is the same across all conditions. As a performance metric, we use the models’ delta log-likelihoods (ΔLL) relative to the baseline model, where the mean log-likelihood of our baseline model per subject was −4991.5 with an SEM of 58.5. The ΔLL values for all models are listed in Table A1 in the Appendix.

**Table 2.** Prior distributions and estimated posterior means with their standard deviations for the Bayesian model parameters are listed in this table. It is to be noted that all Truncated-Normal priors are assigned a lower bound of 0.

Parameter	Prior distribution	Hyperparameters	Posterior mean (SD)
λ	Beta	α = 1, β = 10	0.09 (0.0074)
*p*	TruncatedNormal	μ = 2, σ = 0.1	1.99 (0.091)
*q*	TruncatedNormal	μ = 0.5, σ = 0.3	0.58 (0.023)
β_*s*_	TruncatedNormal	μ = 0.5, σ = 0.25	1.35 (0.155)
σ_*noise*_	TruncatedNormal	μ = 0.1, σ = 0.05	0.023 (0.006)
*b*	TruncatedNormal	μ = 1, σ = 0.5	2.36 (0.556)
*k*_*factor*_ (multiplicative factor)	TruncatedNormal	μ = 1, σ = 0.8	0.65 (0.021)

**Table 3.** Target and normalization pool sizes in various units. We define the size as four times the standard deviation. The normalization pool sizes are extracted from our preferred model. Conversion to cortical extent is based on the equations given by Rovamo et al., (1978).

	Spatial frequency	Target size			Norm. pool		
Eccentricity	cpd	cycles	degrees	mm cortex	Cycles	degrees	mm cortex
Fovea (0°)	1	2	2.0	15.91	—	—	—
	2	2	1.0	7.96	—	—	—
	4	2	0.5	3.98	—	—	—

5°	1	2	2.0	6.74	2.6	2.6	8.76
	2	2	1.0	3.37	2.6	1.3	4.38
	4	2	0.5	1.68	2.6	0.65	2.19

10°	1	2	2.0	4.27	2.6	2.6	5.55
	2	2	1.0	2.14	2.6	1.3	2.78
	4	2	0.5	1.07	2.6	0.65	1.39

**Table A2.** The summary of estimated σ_*n*_ and other parameters against each model is given in this table, where *s*(1, 5) and *a* were fixed to 1 while parameter estimation.

Parameter	separatePool	separatePoolCSF	freqPool	freqPoolCSF	IPLPoolCSF
λ (Lapse rate)	0.075	0.080	0.077	0.100	0.09
*p* (Excitatory exponent)	2.000	2.000	2.000	4.115	2.282
*q* (Inhibitory exponent)	0.715	0.717	0.713	0.529	0.588
σ_*noise*_ (Internal noise)	0.446	0.3801	0.498	0.601	0.614
β_*s*_ (Semisaturation)	0.013	0.621	0.020	0.208	0.839
*b* (Relative scaling)	0.952	11.470	0.987	4.501	5.411
Multiplicative factor (*k*_*factor*_ in Equation 10)	—	—	—	—	0.585
Standard deviation of Normalization Pool (σ_*n*_)					
σ_*n*(1, 5)_ (or σ_*n*1_)	0.525	0.620	0.587	0.306	—
σ_*n*(1, 10)_	0.637	0.236	—	—	—
σ_*n*(2, 5)_ (or σ_*n*2_)	0.319	0.238	0.315	0.221	—
σ_*n*(2, 10)_	0.316	0.325	—	—	—
σ_*n*(4, 5)_ (or σ_*n*4_)	0.149	0.160	0.151	0.142	—
σ_*n*(4, 10)_	0.157	0.175	—	—	
Sensitivities *s*(*f*, *e*)					
*s*(1, 10)	0.591	—	0.869	—	—
*s*(2, 5)	0.646	—	0.818	—	—
*s*(2, 10)	0.493	—	0.616	—	—
*s*(4, 5)	0.485	—	0.589	—	—
*s*(4, 10)	0.243	—	0.323	—	—
Total parameters (*κ*)	17	12	14	9	7

**Table A1.** Comparison of model performance via leave-one-out cross-validation. The ΔLL are estimated relative to the baseline model *fixedPool* (the higher, the better). The SEM are given in parentheses.

	separatePool	separatePoolCSF	freqPool	freqPoolCSF	IPLPoolCSF
Model Variant	($6\;\sigma_{g_{norm}}$)	($6\;\sigma_{g_{norm}}+\text{stelaCSF}$)	($3\;\sigma_{g_{norm}}$)	($3\;\sigma_{g_{norm}}+\text{stelaCSF}$)	(inverse power law + stelaCSF)
Mean ΔLL	36.6 ± 11.0	36.84 ± 10.83	37.0 ± 10.63	34.67 ± 10.53	34.16 ± 11.36
Model Params (*k*)	17	12	14	9	7
Model ΔAIC	−63.21	−73.69	−70.09	−75.35	−78.34

*Note*: Baseline model *fixedPool* mean LL = −4991.5 ± 58.5. The smaller AIC is the better fit model.

#### The separatePool model

We start with the most complex model in which we estimate six different σ_*n*_ values for the six different conditions used in our experiment. This result is referred to as the *separatePool* model. Recall that σ_*n*_ is the standard deviation of the weights of the normalization pool *g*_*norm*_(**x**, σ_*n*_), as defined in Equation 4. The *separatePool* model achieves a ΔLL of 36.6 with a standard of the error mean (SEM) of ± 11.0 and a change in the Akaike information criterion (ΔAIC) of −63.21 relative to the baseline model. Note that a smaller AIC indicates a better trade-off between model fitting and model complexity. Indeed, this model successfully fits our psychophysical data (see the Appendix, Figure A1).

**Figure 4.**
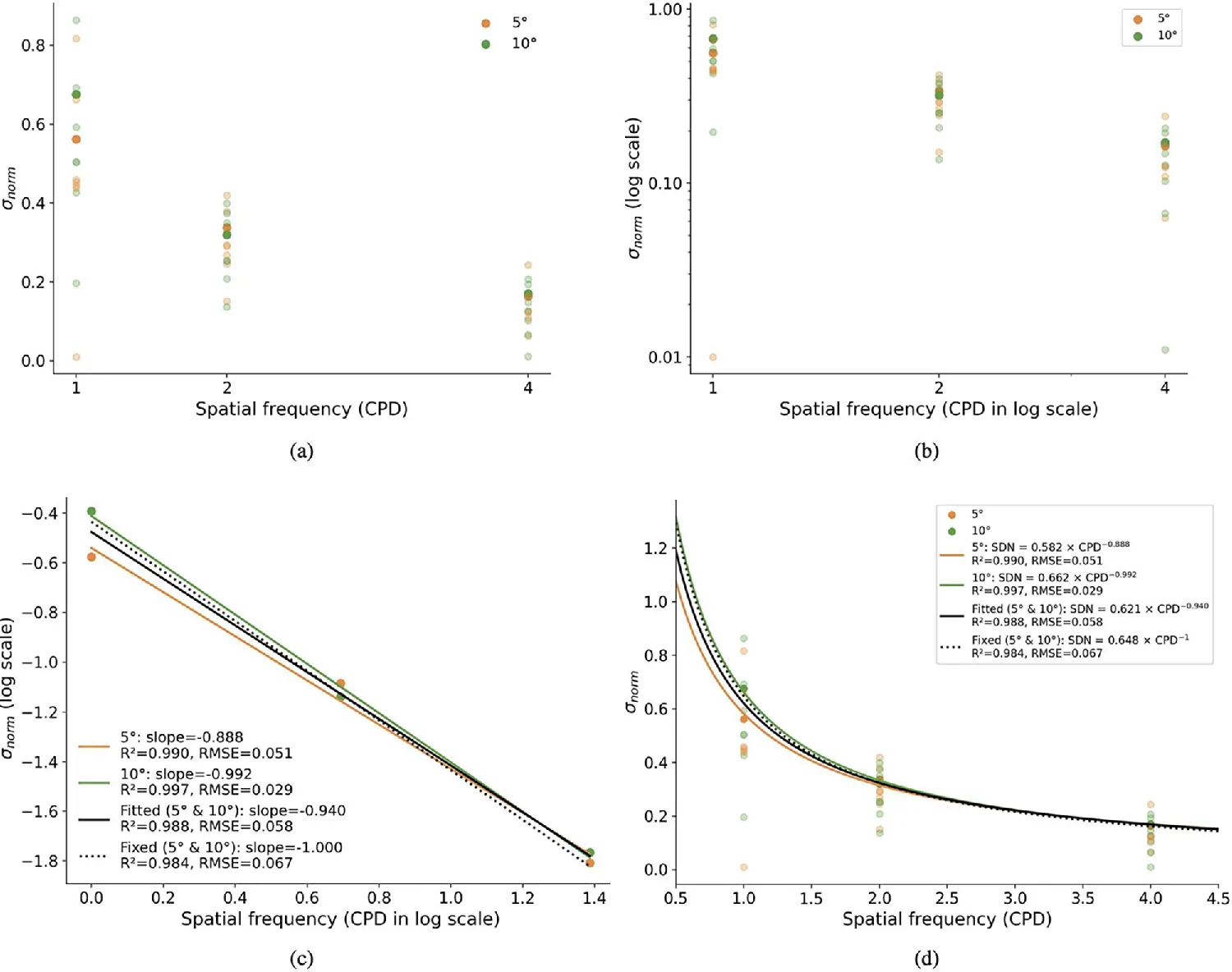
(a) The standard deviation σ_*n*_ is plotted against three different cpds, where the orange and green lines represent 5° and 10° eccentricities, respectively. Different eccentricities have minimal effect on σ_*n*_ as they are almost overlapping each other. (b) Both the *y*-axis and the *x*-axis are converted to log domain. (c) To study the relationship between the normalization pool size and the spatial frequencies, linear regression is applied to estimate the slope and intercept. (d) The relationship between cpd and σ_*n*_ is a power law function with exponents of −0.888 and −0.992; the constants are 0.582 and 0.662 for the 5° and 10° eccentricities, respectively.

**Figure 5.**
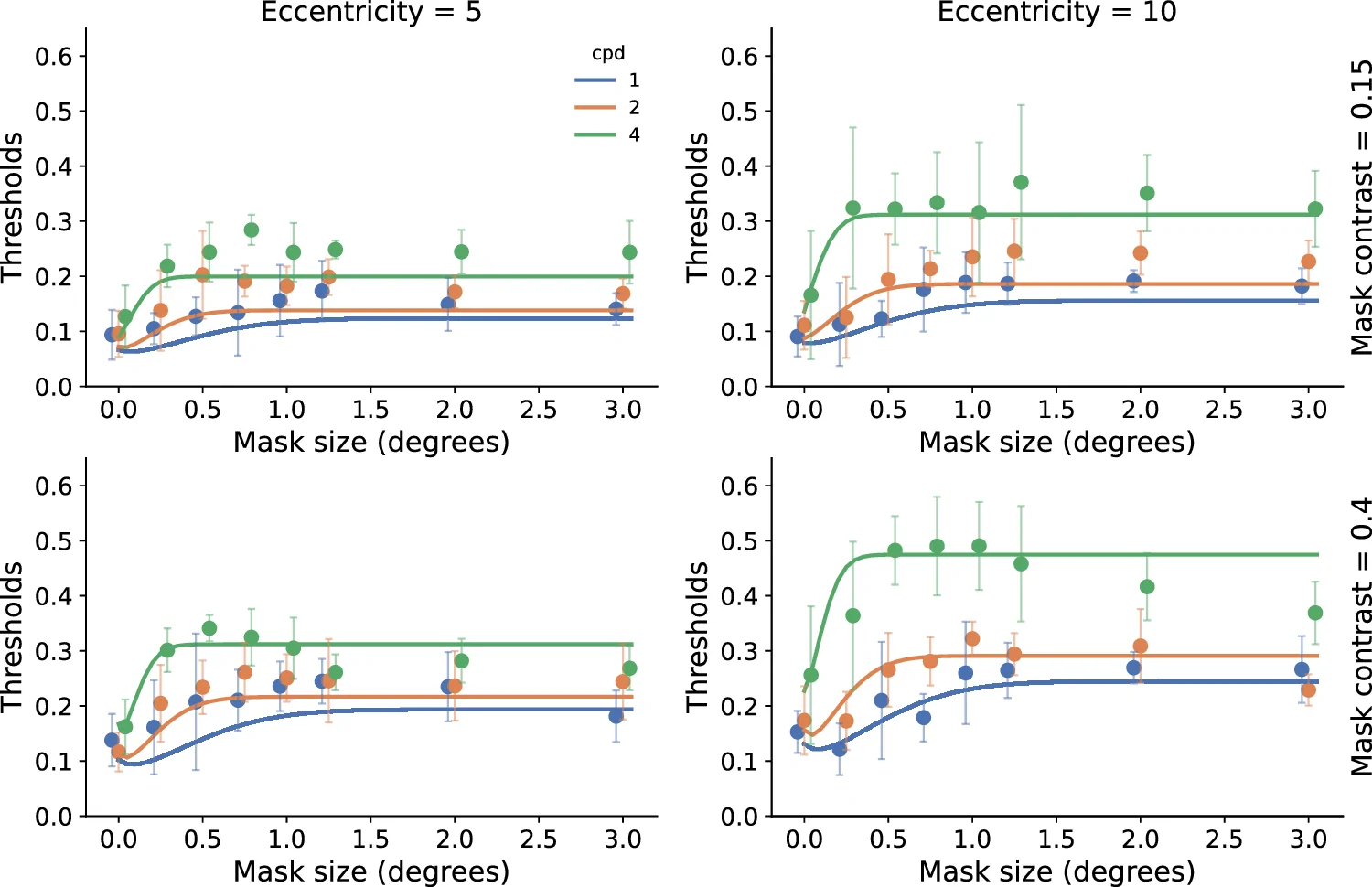
In these plots, the estimated response of our preferred model titled *IPLPoolCSF* is represented as solid curves in each subplot based on the combination of eccentricity and mask contrast. The data points represent the averaged response of the seven observers and the error bars represent 95% confidence intervals.

**Figure 6.**
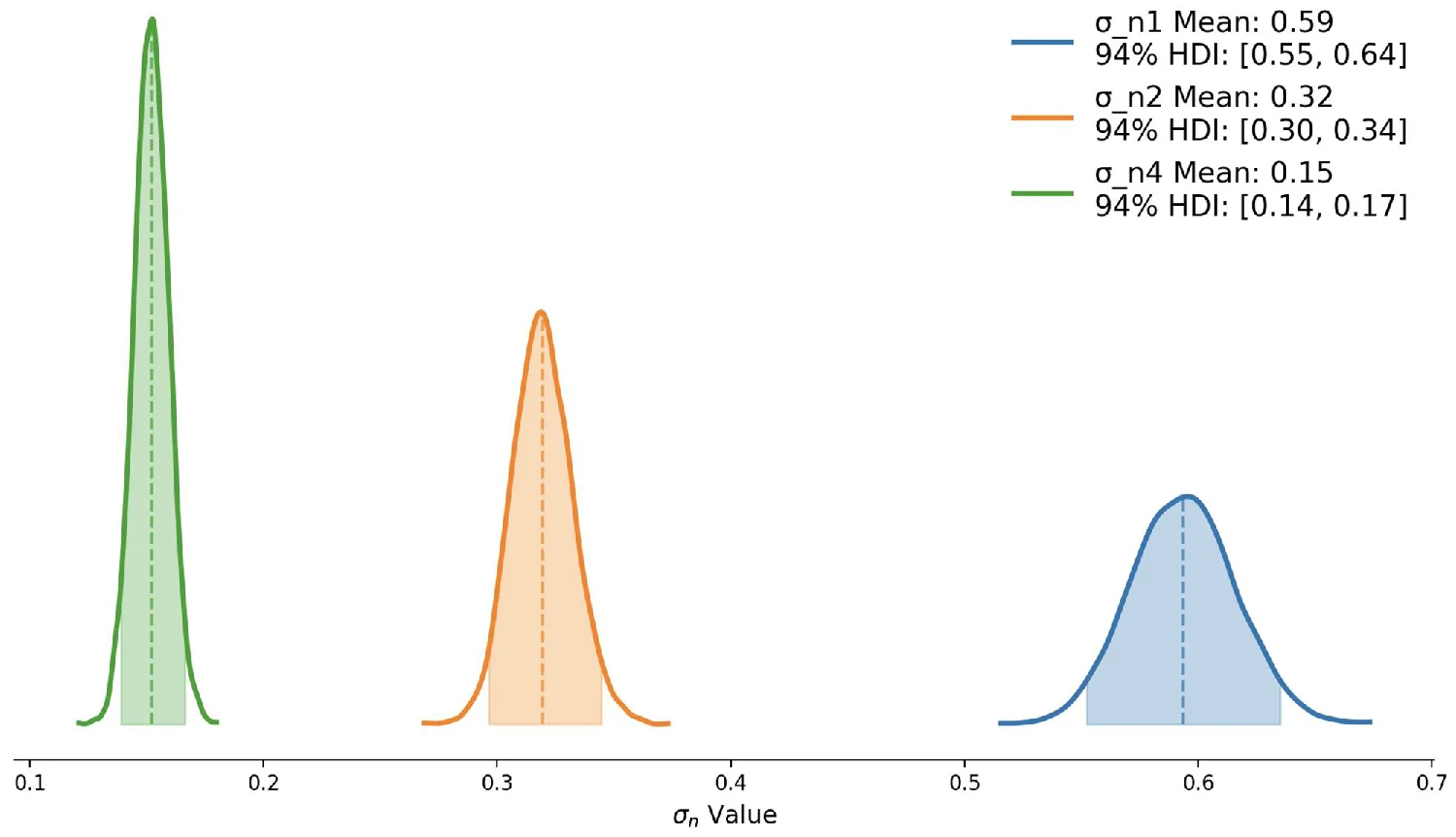
The plot shows nonoverlapping posterior distributions of three σ_*n*_ values in the Bayesian version of our *freqPool* model (see the Appendix), verifying that the normalization pool size is not fixed but scales inversely with the spatial frequency of the stimulus. HDI, highest density interval.

**Figure 7.**
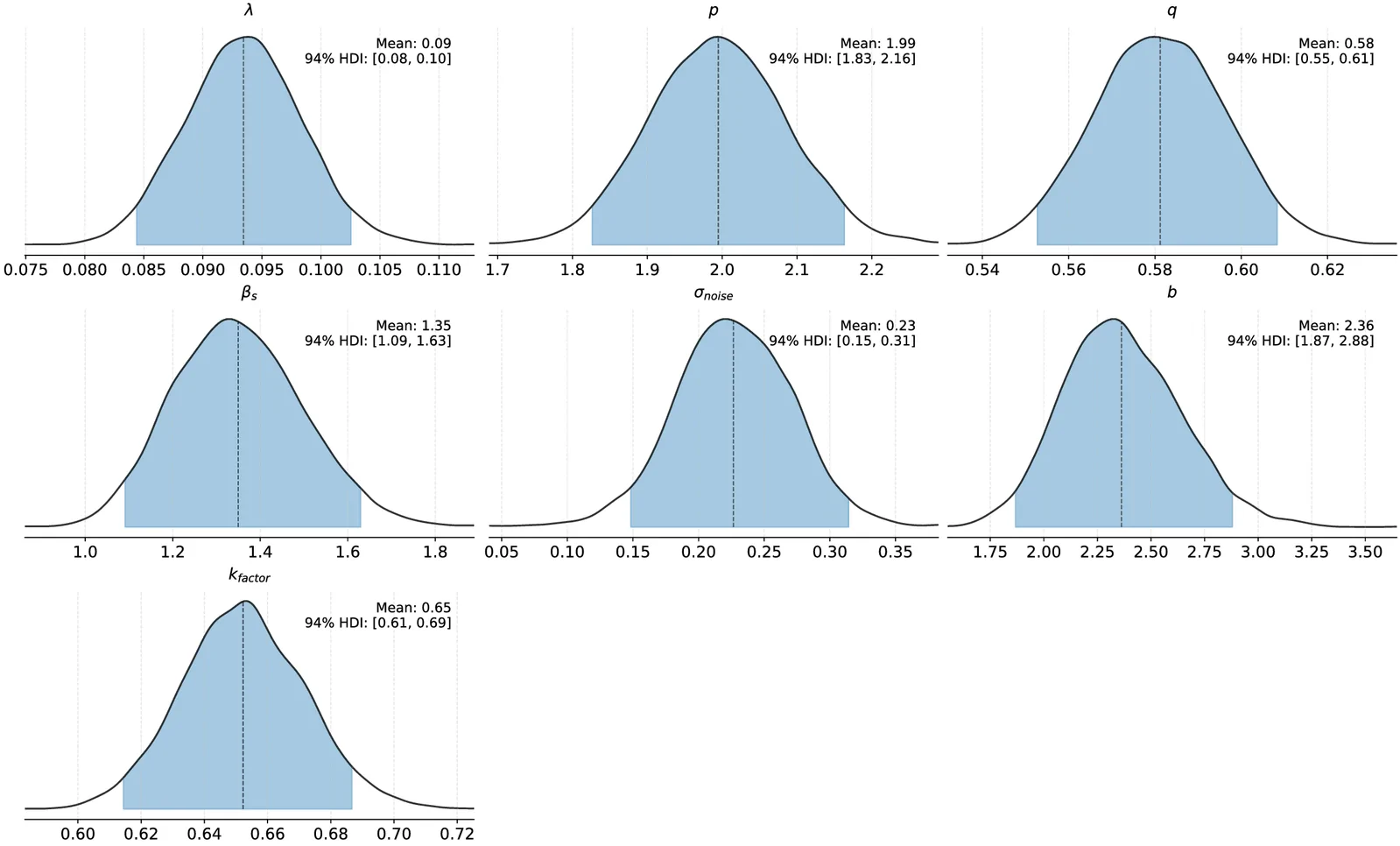
The marginal posterior distributions for the parameters of the preferred model (*IPLPoolCSF_Bayesian*) are shown in this figure. Each subplot shows the parameter density, posterior mean (dashed vertical line), and 94% highest density interval (HDI, blue-shaded region).

**Figure 8.**
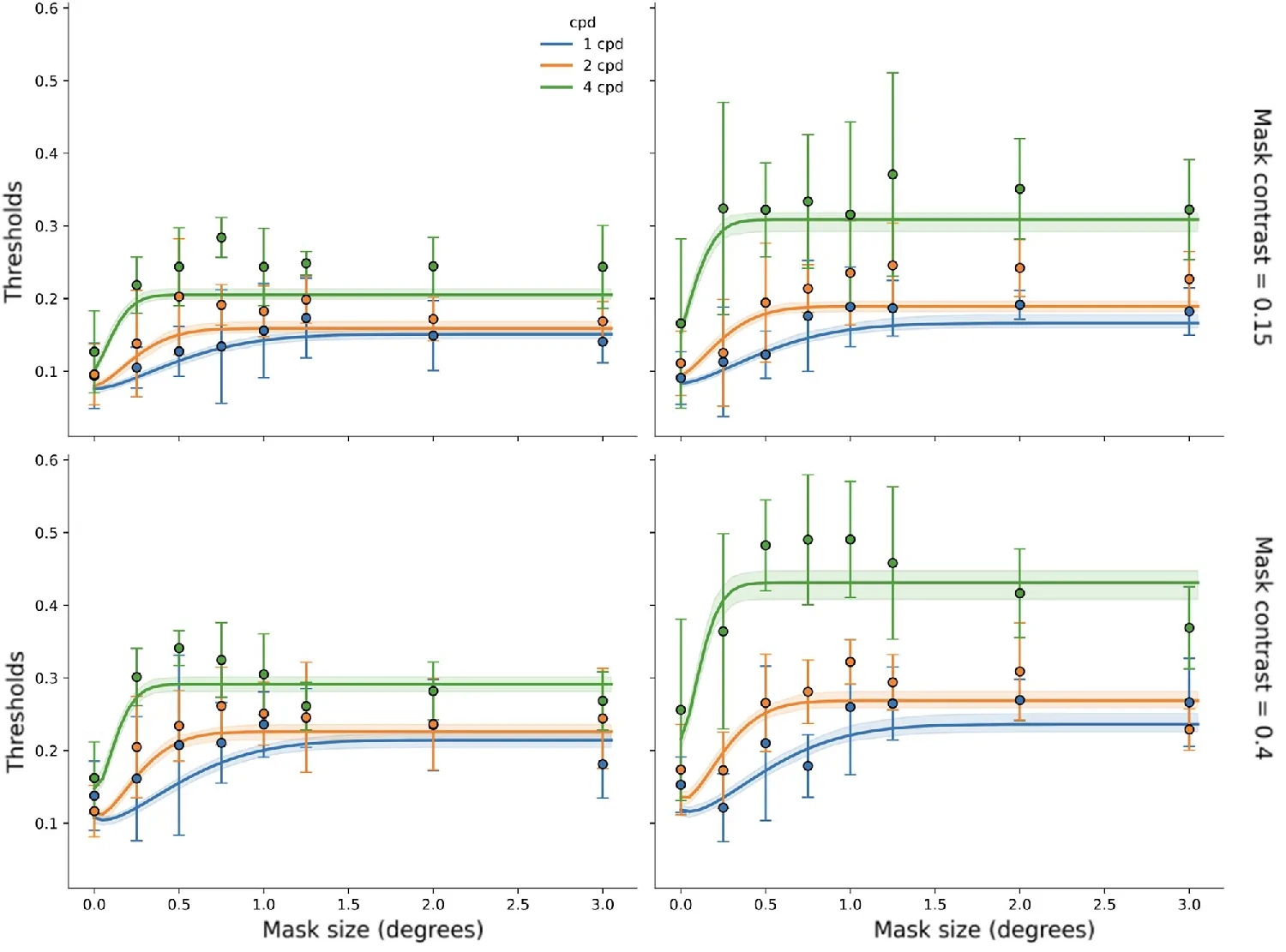
The shaded region presents responses from the posterior predictive analysis based on 50 different sets of model parameters sampled from the posterior distributions. The solid lines represent the mean of these 50 predicted responses across these parameter sets, and the dots represent the actual observers’ data. The model successfully captures the threshold saturation and the dependence on mask size across eccentricities (5° and 10°) and spatial frequencies.

**Figure A1.**
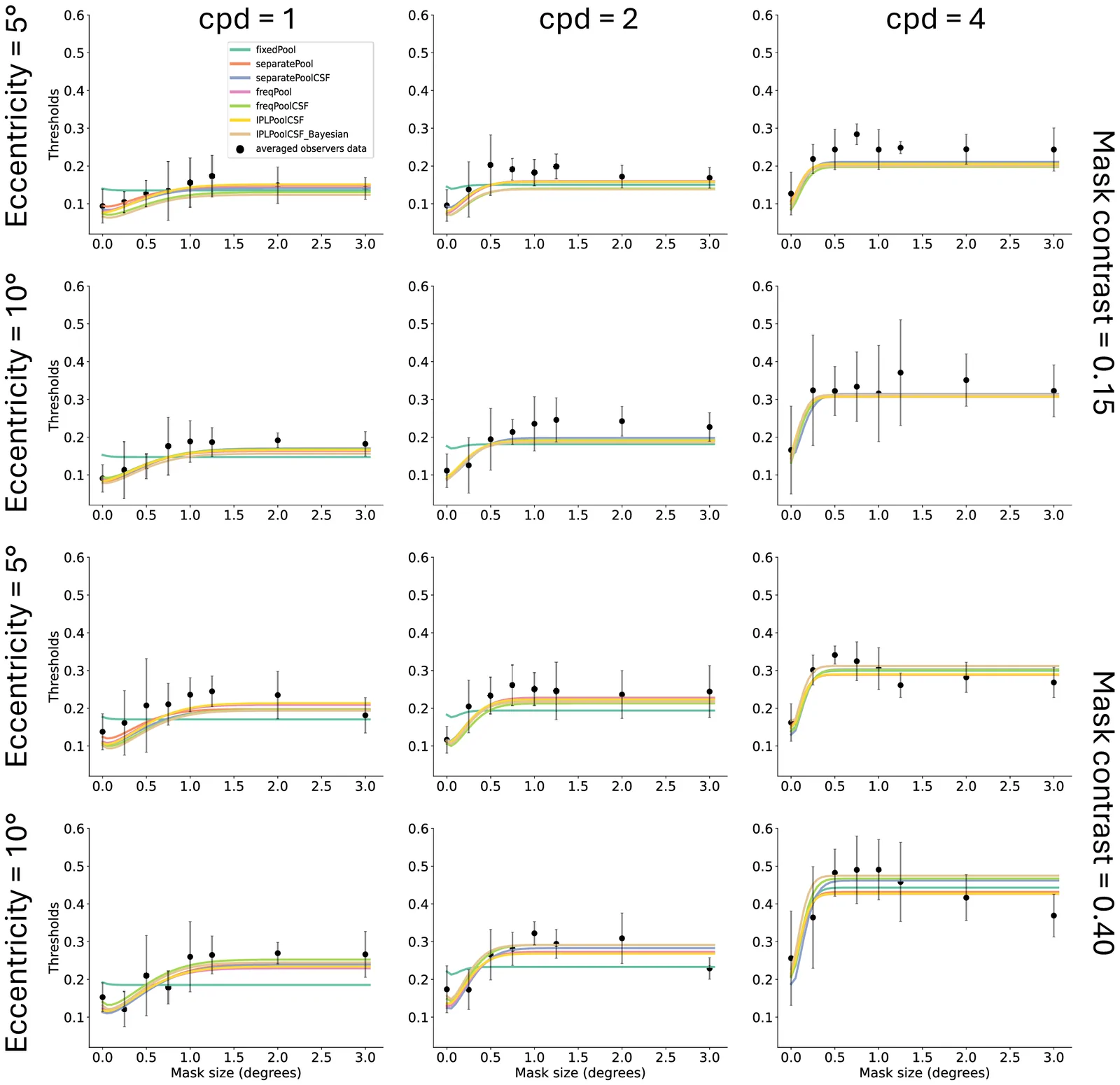
The estimated response of each model is represented within each plot. The data points represent the averaged response of the seven observers, and the error bars are the 95% confidence intervals. Among the different model responses, it can be observed that the Bayesian version of *IPLPoolCSF* performs well in capturing the actual observers’ data, which are represented as black dots.

Additionally, we performed a parameter recovery analysis for the *separatePool* model to confirm that the model parameters, especially the six σ_*n*_ values, can be estimated reliably from a finite data set comparable in size to our experiment. The reason for using the *separatePool* model for model recovery analysis is that successful recovery of this most complex model would imply recoverability for the simpler models as well. We generated 100 synthetic data sets using the 17 fitted *separatePool* model parameters while retaining the original trial-by-trial target contrasts and replacing only the observed correctness values with binary responses sampled from the model. Each experiment followed the same experimental design, where 7 observers performed 96 peripheral conditions with 100 trials per block. The model was then refitted using the same likelihood, initializations, bounds, and optimization framework as in training the model on actual observer data. The six σ_*n*_ values were recovered with absolute relative bias ranging from 1.7% to 3.3%, and relative root mean square error (RMSE) ranged from 6.2% to 13.7%. The relative RMSE was below 7% for the pool sizes of 1 cpd and 2 cpd, but was higher for the normalization pool’s σ_*n*_ values of 4 cpd because a small number of fits converged to atypical solutions. The logarithmic slopes that defined the relationship of the size of the normalization pool with spatial frequency were recovered with a relative bias of less than 1% and a relative RMSE of 7.6%. These results validated that the size of the normalization pool and its inverse relationship with spatial frequency can be reliably estimated from data sets of the size used in our experiment. The logarithmic slopes that define the relationship between the size of the normalization pool and the spatial frequency were recovered with absolute relative biases of 3.0% and 2.4% at 5° and 10°, respectively, and relative RMSE values of 13.0% and 12.3%, respectively. Thus, we expect our estimates of the normalization pool sizes to be reasonably accurate with a relative RMSE of about 10%.

#### Preferred model

Although the *separatePool* model performs well, we wanted to find a model that does not require condition-specific parameters, such that we can make predictions for conditions that did not appear in our experiment. An analysis of the parameters of the *separatePool* model, as shown in Figure 4, revealed that σ_*n*_ was largely independent of eccentricity (5° vs. 10°) as the estimates were highly overlapping with each other, as shown in Figure 4a. Therefore, we constrained the model to use the same parameters for both eccentricities.

Towards-modeling the effect of spatial frequency on the size of the normalization pool, we performed a linear regression on their logarithms, as shown in Figure 4c. Hence, we found that the relationship between the spatial frequency and the size of the normalization pool (σ_*n*_) is a power law function which can be defined by Equation 10 with fitted exponents of −0.888 and −0.992, and *k*_*factor*_, that is, multiplicative factors of 0.582 and 0.662 for 5° and 10° eccentricities, respectively, as depicted in Figure 4d.

(10) $$\begin{array}{c} \sigma_{n}=k_{factor}\times f^{-1}\; \end{array}$$

These parameter estimates are very similar, which again hints that eccentricity does not have a large effect on the normalization pool, and the exponents are close to −1. Hence, we incorporated a power law relationship between the spatial frequency and the normalization pool size σ_*n*_ with the exponent fixed to −1, representing a strict inverse law with a shared scaling factor (*k*_*factor*_) for 5° and 10° eccentricities. Additionally, we integrated stelaCSF to reduce the number of parameters to be estimated as shown in Equation 9. Hence, the sensitivities with respect to spatial frequencies and eccentricities are finally extracted from the stelaCSF instead of directly estimating these values during model fitting.

The mean ΔLL achieved by this final best model is 34.6 with an SEM of ± 10.53. Here, ΔLL is only 2 points below the most complex model (i.e., *separatePool* model) with 10 fewer parameters and a ΔAIC of −78.35 relative to the baseline model as given in Table A1, where a smaller AIC indicates a better trade-off between model fitting and model complexity. This model fits the threshold data from our observers very well (see Figure 5). We have referred to this final best model as *IPLPoolCSF* in the Appendix. The intermediate models’ predictions are similar to this model (see Appendix Figure A1), and none of the estimated parameters disagree with the conclusions drawn based on it (see Table A2 given in the Appendix).

To verify the effect of the variability being approximately proportional to the mean, we modified Equations 5 and 6 and retrained our final preferred model named *IPLPoolCSF*. After fitting the model and performing cross-validation, it was found that the ΔLL improved from 34.169 to 36.174, and the ΔAIC value improved from −75.3 to −80.3. Because the results have not changed significantly, we decided to keep the results based on the assumption that the noise variance is constant.

### Bayesian modeling

We implemented a Bayesian version of our preferred model to validate the robustness of our maximum likelihood estimates and to quantify the uncertainty of the model parameters. This approach allows us to incorporate prior knowledge about biologically plausible parameter ranges and quantify the uncertainty by obtaining full posterior distributions for our model parameters.

#### Implementation and sampling

The probabilistic model was constructed using the PyMC library in Python (Abril-Pla et al., 2023). Our generative model predicts the probability of a correct response based on the stimulus parameters, where the observed correctness is modeled as a Bernoulli trial. We assigned weakly informative truncated normal priors to all parameters except λ to constrain them to positive, biologically plausible values (refer to Table 2). We sampled the posterior distribution using the no-U-turns Sampler with 4 independent Markov chains, each with 750 tuning steps and 1,500 draw iterations (6,000 total samples). Convergence was confirmed using the Gelman–Rubin statistic ($\hat{R}\approx 1.00$) and effective sample size (>2,400) for all parameters.

#### freqPool model

For Bayesian analysis, we implemented a model with separate estimates of the size of the normalization pool for each spatial frequency, which is referred to as *freqPool* model (see the Appendix for details). For this particular model, the posteriors for the normalization pool sizes are quite narrow and do not overlap with each other, as shown in Figure 6. The clear separation of these distributions confirms that our experimental data require the normalization pool to change size with the spatial frequency. Strict inverse scaling with the spatial frequency also fits the relative position of the posteriors well.

#### Preferred model

The Bayesian estimates of the *IPLPoolCSF* model parameters align well with theoretical expectations as given in Table 2 and Figure 7, where all parameters appear to be well constrained by the data. For example, the estimated exponent *p* ≈ 1.99 matches the squared-energy model standard in visual neuroscience. The posterior predictions of the Bayesian version of the final model with a fixed exponent equal to −1 align well with the observers’ true threshold values and capture the dependence on mask size at spatial frequencies, as shown in Figure 8. The close alignment between the predicted thresholds and the observed data confirms that a single normalization pool scaling factor (*k*_*factor*_ ≈ 0.65), inversely proportional to spatial frequency, is sufficient to explain the masking effects across all eccentricities. Please note that it is referred to as *IPLPoolCSF_Bayesian* in Figure A1.

## Discussion

We conducted a 2AFC contrast masking experiment to measure the spatial extent of the normalization pool while varying the eccentricity along the horizontal meridian, spatial frequency, and contrast. We found categorically different behavior in the fovea and in the two peripheral locations: In the fovea (0° eccentricity), the mask size did not affect the contrast thresholds, although the detection thresholds were clearly elevated and increased further at higher mask contrast. In the periphery, we found a gradual increase in contrast thresholds with mask size, which was well fitted by a divisive normalization model (Carandini & Heeger, 2012). The spatial extent of the normalization pool is inversely proportional to the spatial frequency, corresponding with a standard deviation of 0.65 cycles, regardless of contrast and retinal location.

For the periphery, behavioral data are well fitted by a divisive normalization model, where the normalization pool standard deviation σ_*n*_ is 0.65° for 1-cpd channels and scales inversely with the spatial frequency to 0.32° at 2 cpd and 0.16° at 4 cpd using Equation 10. This result is consistent with a scale-invariant account of visual processing, where the receptive field size is inversely proportional to the frequency, such that it covers an equal number of cycles and the receptive fields at different frequencies are scaled versions of each other. The fact that the normalization pools do not vary significantly between eccentricities strongly hints against the scaling of the normalization pool with cortical magnification as these two eccentric locations, 5° and 10°, differ in cortical magnification by a factor of about 1.5 (Rovamo et al., 1978). Therefore, this should have resulted in an easily detectable difference between locations.

Our measurements align well with the limited physiological data available in the literature related to the normalization pool. Cavanaugh et al. (2002b) estimated that the nonclassical receptive field is about three times the size of the classical receptive field based on electrophysiological measurements. If we assume that classical receptive fields are scaled versions of each other, that would correspond with to the normalization pool scaling inversely proportional to the spatial frequency, as we observed here. Also, in absolute terms, neurons the in the V1 have typical bandwidths of 1.4 octaves for simple cells and 1.5 octaves for complex cells according to physiological data (De Valois et al., 1982). These bandwidths correspond with a standard deviation of 0.344° for the classical receptive field of a 1-cpd neuron. Our estimated pool size of 0.65° at 1 cpd is 1.8 times greater than this value (refer to Table 3). Although this value is lower than the physiological measurement for an average neuron, it remain within the range of reasonable normalization pool sizes. (Cavanaugh et al., 2002b). However, a slight complication of this explanation is that the bandwidth of neurons varies with spatial frequency according to physiological data (De Valois et al., 1982), so that the relationship between the size of the receptive field and the spatial frequency is shallower than the strict inverse scaling with spatial frequency. According to De Valois et al. (1982), the mean spatial frequency bandwidths are about 1.8, 1.6, and 1.5 octaves for 1-, 2-, and 4-cpd neurons, respectively. When converting these values into standard deviations of the classical receptive field in visual degrees, they are mapped to 0.28°, 0.16°, and 0.08° with respect to 1, 2, and 4 cpd. This corresponds with a slightly shallower decrease in the spatial frequency than inversely proportional scaling. To match the larger receptive fields, the exponent should have been log (0.08/0.28)/log (4) = −0.90 instead of −1, which we cannot exclude on the basis of our data. Given the substantial variability of receptive field sizes in the physiological data, the measurement error on our data, and the potential differences between humans and macaques, the agreement of our results with physiology seems quite aligned.

For the fovea, we do not observe an effect of the mask size on the contrast thresholds. This is in contrast with the two eccentric conditions, which are well fitted with the same normalization pool sizes. We found four potential explanations for such a small pool and the lack of a mask size effect in the fovea. First, the fovea might be inherently specialized; for instance, its normalization pool might be small enough that the target envelope already covers it entirely. Another possibility is that detection is limited by noise from areas beyond the V1, where the spatial extent of receptive fields and normalization pools differs.

Second, one could suspect a task effect. We presented the stimuli consecutively in the foveal condition, while they were presented simultaneously in the peripheral condition. That should not make a difference in the threshold patterns as two alternative and two interval forced choice experiments generally yield equivalent results. To verify this, we performed a short control experiment with the last author as the observer to compare the 2AFC and 2IFC tasks. We compared the two tasks at two mask sizes with 200 trials per condition at 5° eccentricity with 2 cpd and a 40% mask contrast. We showed targets on both sides of fixation in the 2IFC task to reduce involuntary eye movements toward the stimuli and used the same temporal structure as for the foveal task. The thresholds obtained with the 2IFC task were almost twice as high as those obtained with the 2AFC task, indicating that the 2IFC paradigm was more difficult. However, both tasks show a similar increase in thresholds for the larger mask size, confirming our most basic finding for the periphery with a 2IFC task. Thus, it seems that we can exclude this potential reason for the difference between the fovea and the periphery.

Third, our stimuli could be of too low a spatial frequency for the fovea. Below some retinal location-dependent spatial frequency, neurons in the V1 become low pass instead of bandpass (Ringach, 2002), and psychophysical experiments do not find evidence for low spatial frequency channels either (Blakemore & Campbell, 1969). These low-pass neurons have smaller receptive fields than one would otherwise expect for the low spatial frequencies and could have particularly small normalization pools too, which could explain our observations. This explanation does not fit some other observations well, though. To explain the lack of effect at 4 cpd, this explanation requires a surprising distribution of neurons in the fovea. Blakemore and Campbell (1969) found that the lowest spatial frequency channel in the fovea has a peak sensitivity at 3 cpd. Additionally, cortical magnification suggests that the 1-cpd targets at 5° eccentricity subtend a larger area in cortex than the 4-cpd ones in the fovea. Thus, if there were no neurons tuned to 4 cpd in the fovea we would not expect neurons tuned to 1 cpd at 5° eccentricity either.

Fourth, the spatial gradient of sensitivity could be so large in and near the fovea that the outer range of the normalization pool is substantially less sensitive than the center where the target is located. In other words, there is nearly no normalization effect in the fovea. This could make the normalization pool seem small, potentially explaining our results.

Future experiments with stimuli placed between 0° and 5° eccentricity with higher spatial frequencies should enable distinction between our different hypotheses. Unfortunately, data for small-sized, high spatial frequency targets close to the fovea are hard to collect both psychophysically and physiologically. For such experiments, fixation and eye movements become important influences, accurate display of stimuli is hard, stimuli become hard to detect, and stimuli drive neurons less and less. However, such experiments could be worthwhile extensions for future studies.

Including our results in image-computable models (Hore & Ziou, 2010; Laparra, Muñoz-Marí, & Malo, 2010; Mantiuk et al., 2024; Schütt & Wichmann, 2017) would be relatively straightforward for both the foveal and the peripheral results we obtained. To incorporate our findings as we found in the fovea, we would make the normalization perfectly local, which is aligned with how current models implement normalization pooling. To enable models to yield results as we observe for the peripheral locations, they would require one additional convolution to compute the activity in the normalization pool, where the size of the pooling kernel can be computed directly from our fitting results. The relationship we observed would be particularly easy to implement in models based on pyramid decompositions (Simoncelli, Freeman et al., 1995), where it corresponds with equally sized normalization pools in terms of pixels in the representation for the different frequency bands.

The findings of our study would contribute to the development of better low-level vision computational models through behavioral evidence on the spatial extent of the normalization pool that satisfies physiological scaling (Cavanaugh et al., 2002a; Cavanaugh et al., 2002b). In addition, these findings could potentially improve image quality assessment metrics (Laparra et al., 2010; Hore & Ziou, 2010; Mantiuk et al., 2024) and enhance the robustness of biologically inspired deep neural networks (Dapello et al., 2020; Geirhos et al., 2018; Kruger et al., 2012; Wichmann & Geirhos, 2023).

## Appendix

### Model comparison

We initially compared six different variants of our proposed model across all conditions, where each condition consisted of six combinations using three spatial frequencies (1, 2, and 4 cycles per degree) and two eccentricities (5° and 10°). Varying the mask contrast (15% vs 40%) had no significant effect on the normalization pool. Note that the foveal data were intentionally excluded from the modeling analysis because the mask size has a minimal effect on these thresholds. Table A2 summarizes the estimated parameters against each model. The six variants of our proposed model are listed below:

• *fixedPool* serves as the baseline in which we consider that the size of the normalization pool does not vary under different stimulus conditions; for example, the size of the normalization pool is the same across all conditions.
• *separatePool* is the most complex model in which we estimate six different σ_*n*_ values for the six different combinations of spatial frequency and eccentricity.
• *separatePoolCSF* integrates stelaCSF into the *separatePool* model to reduce the number of parameters to be estimated as shown in Equation 9.
• *freqPool* estimates three σ_*n*_ values (one per spatial frequency), sharing parameters between eccentricities.
• *freqPoolCSF* incorporates stelaCSF with the *freqPool* model.
• *IPLPoolCSF* is defined with a fixed exponent of −1 as a strict inverse power law given in Equation 10.

The estimated responses of these models are shown in Figure A1. It can be easily observed from these graphs that the *separatePoolCSF* model with (6 σ_*n*_) performed better than the *separatePool* and models in capturing the actual observer’s response. Although *separatePool* model performed well, an analysis of its estimated parameters in Figure 4 revealed that σ_*n*_ is largely independent of eccentricity (5° vs. 10°). Therefore, we constrained the eccentricities to share parameters, resulting the *freqPool* model and the *freqPoolCSF* model. For model selection, we performed leave-one-out cross-validation and compared their ΔAIC values, which are summarized in Table A1. The most simplified *fixedPool* model is considered as our baseline. The mean log-likelihood of this baseline model is −4991.5 with a standard error mean of ±58.5. Considering the mean Δ log-likelihood ± SEM and the ΔAIC relative to the baseline model, we found that, *IPLPoolCSF* model performed best among these variants. Hence, it is considered as our preferred model, which incorporates the inverse power law relationship between spatial frequency and normalization pool size; it also includes stelaCSF.

Furthermore, to study the effect of the spatial frequency on the size of the normalization pool through an analysis of the estimated parameters of model *separatePool* (6 σ_*n*_), we applied linear regression to estimate the slope and intercept on a logarithmic scale, as shown in Figures 4b and 4c. The relationship between the spatial frequency and the size of the normalization pool (σ_*n*_) is a power law function with exponents of −0.888 and −0.992, and multiplicative factors of 0.582 and 0.662 for 5° and 10° eccentricities, respectively, as depicted in Figure 4d. Hence, we defined the *IPLPoolCSF* model with a fixed exponent of −1 as a strict inverse power law where $\sigma_{g_{norm}}\propto 1/f$, with a single global scaling factor. This model is found to be the preferred model based on the lowest ΔAIC value of −78.34 relative to the baseline model.
